# Supplementary figures and images for: Utilization of screening services on cervical cancer and associated factors among female health workers in Addis Ababa, Ethiopia
Source: PLoS One. 2025 Apr 21;20(4):e0321662. doi: 10.1371/journal.pone.0321662 (PMC12011226; doi:10.1371/journal.pone.0321662)

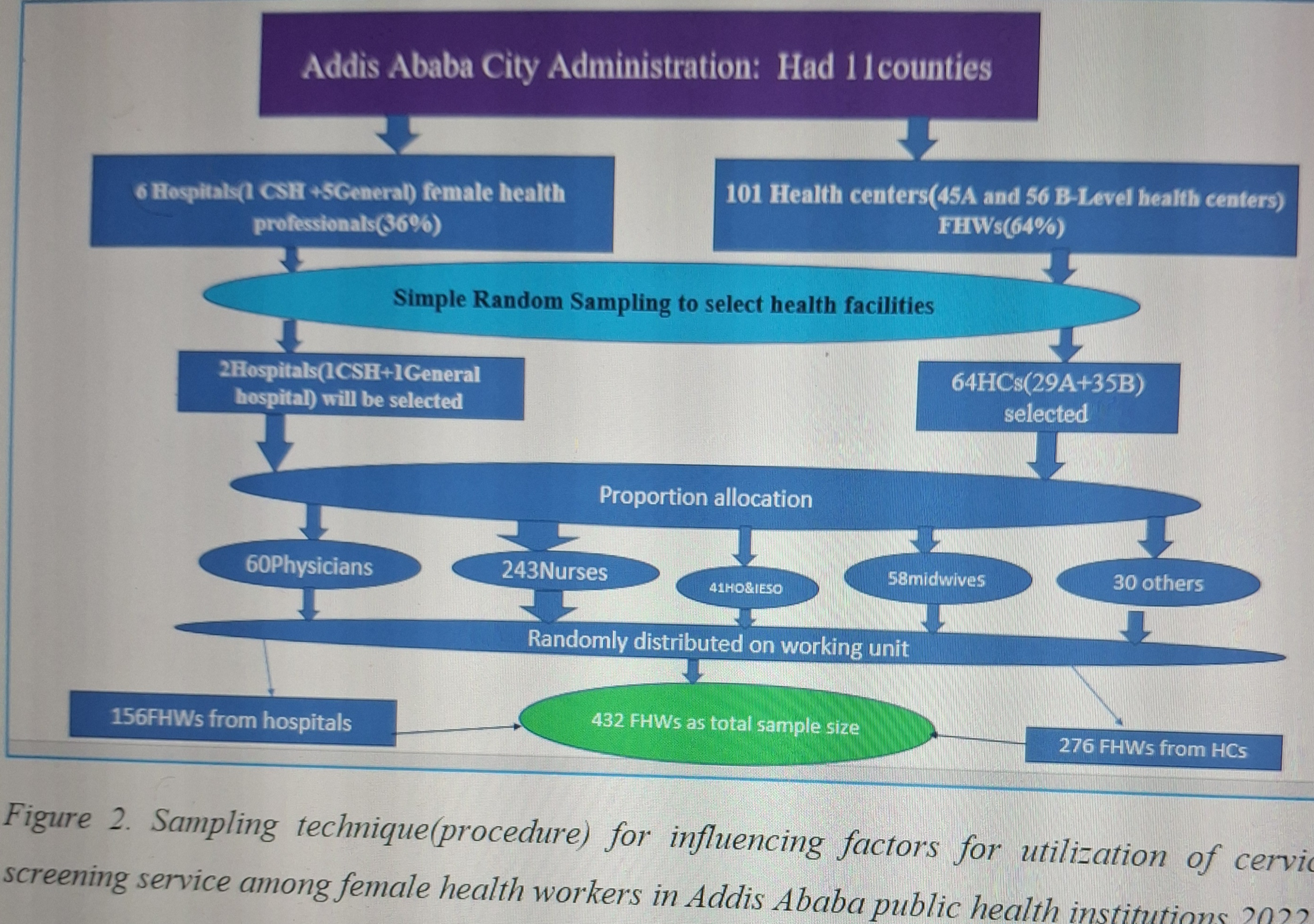

Supplement: S1 Fig — (TIF) [file pone.0321662.s001.tif]

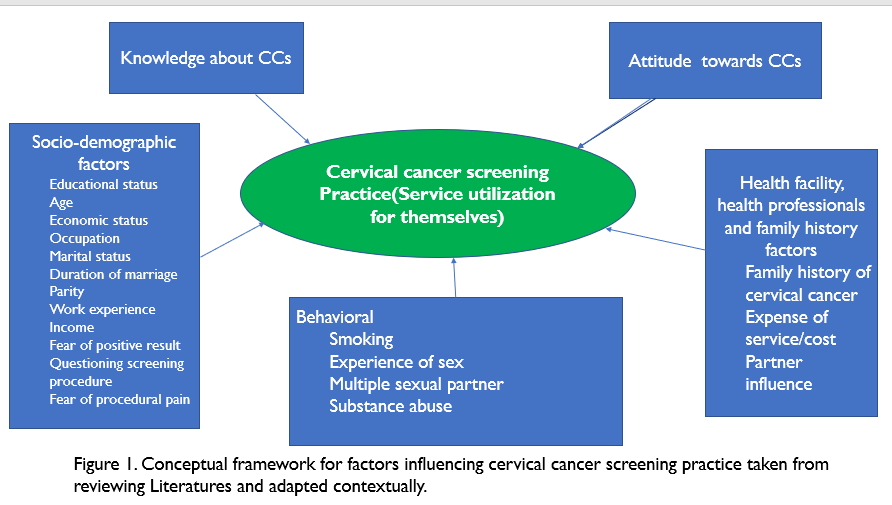

Supplement: S2 Fig — (TIF) [file pone.0321662.s002.tif]

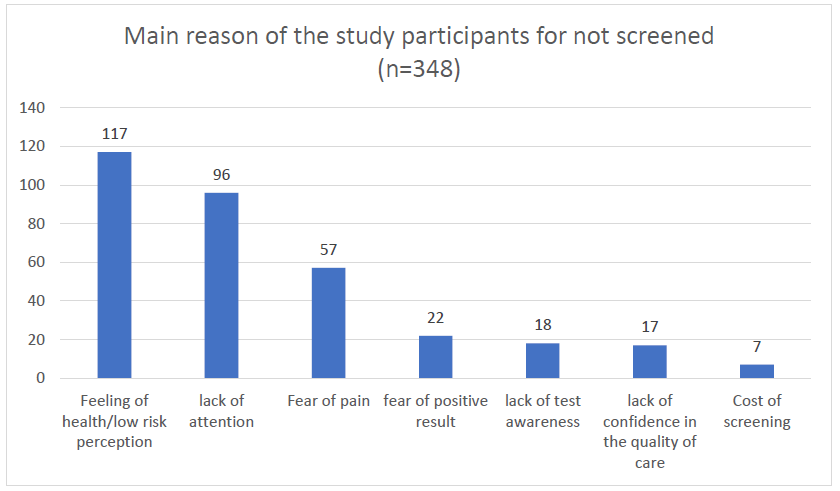

Supplement: S3 Fig — (TIF) [file pone.0321662.s003.tif]
