# Supplementary material for: Utilization of screening services on cervical cancer and associated factors among female health workers in Addis Ababa, Ethiopia
Source: PLoS One. 2025 Apr 21;20(4):e0321662. doi: 10.1371/journal.pone.0321662 (PMC12011226; doi:10.1371/journal.pone.0321662)
Supplement: Attachment — Submitted filename: Response to Reviewers.docx [file pone.0321662.s005.docx]

**Response to Reviewers**

**To**: Muhammad Farooq Umer, PhD Epidemiology and Health Statistics
Academic Editor
PLOS ONE

**Re**: Point-by-point response to the editor’s and reviewers’ comments.

**Title**: “Utilization of screening services on cervical cancer and associated factors among female health workers in Addis Ababa, Ethiopia,”

PONE-D-24-37781.

Dear academic editor and reviewers, we are indebted for taking your time to review our manuscript and providing us with constructive comments.

I. **Response to the editor**.
1. **Comment**: Please ensure that your manuscript meets PLOS ONE's style requirements, including those for file naming.

**Response**: We have carefully and systematically revised our manuscript to meet the PLOS ONE’s style requirements as advised. File naming and formatting has been addressed. The supporting information documents have been included at the end of the main manuscript.

2. **Comment:** Thank you for stating the following financial disclosure:

“This manuscript was partially funded by the Addis Ababa University Office for Graduate studies. The rest of the fund was equally shared among the authors.”

Please state what role the funders took in the study.  If the funders had no role, please state: "The funders had no role in study design, data collection and analysis, decision to publish, or preparation of the manuscript."

If this statement is not correct you must amend it as needed.

Please include this amended Role of Funder statement in your cover letter; we will change the online submission form on your behalf.

**Response**: The funder for this manuscript, Addis Ababa University Office for Graduate Studies, had no role in study design, data collection and analysis, decision to publish, or preparation of the manuscript. This statement is included in the cover letter as per your recommendation.

3. **Comment:** Please ensure that you refer to Figures 1 and 2 in your text, if accepted, the production will need the reference to link the reader to the figure.

**Response:** Thank you for the constructive comment. The figures 1 and 2 were uploaded as supporting information. So, the figures are renamed as S1 Fig and S2 Fig in the text and necessary correction also made in the uploaded supporting information section.

**4.** **Comment**: We note that your Data Availability Statement is currently as follows: “All relevant data are within the manuscript and its Supporting information files.

**Response:** Thank you once again for your comment and clarification. I confirm that all the necessary raw data are within the manuscript satisfying the Plos criteria that “authors must share the minimum set of data”. We have also added an additional raw data that clarifies some of the comments addressed by the editor and the reviewer.

**5. Comment from editor**: When completing the data availability statement of the submission form, you indicated that you will make your data available on acceptance. We strongly recommend all authors decide on a data sharing plan before acceptance, as the process can be lengthy and hold up publication timelines. Please note that, though access restrictions are acceptable now, your entire data will need to be made freely accessible if your manuscript is accepted for publication. This policy applies to all data except where public deposition would breach compliance with the protocol approved by your research ethics board. If you are unable to adhere to our open data policy, please kindly revise your statement to explain your reasoning and we will seek the editor's input on an exemption. Please be assured that, once you have provided your new statement, the assessment of your exemption will not hold up the peer review process.

**Response:** Thank you for your comment and advice. Dear editor (s) as stated above in the response to comment number 4, we have made no restriction to the data availability and we made all the necessary data available in the manuscript submitted. Our submission contains all the raw data to replicate the results of our study. The values behind the mean, standard deviation is presented in the tables which are incorporated in the manuscript. We have also added an additional Figure as supporting information that will complement the previously shared data.

II. **Response to reviewer #1.**

Dear reviewer, we are truly grateful to your rigorous review process to make our manuscript more clear, readable, and consistent. We have seriously considered all your valuable comments and maximum effort was made to incorporate all the comments in the main manuscript. For the details of the replies please refer to the individual point-by-point responses. Please note that the previous “line numbers” defined by the reviewer during the review process may not be maintained through the responses to the comments as there are revisions made in each section of the manuscript starting from the Title page.

1. **Comment:** Line #1: which term is appropriate “utilization” or “use”

**Response**: Thank you for the question. While it seems like a minor distinction, “**use”** is simply the act of using something, while “**utilization**” connotes using something to its best advantage. So, we think keeping the word “utilization” in the topic and the rest of the manuscript will better describe the intent of the study.

2. **Comment**: Line 22: what is the importance of depicting date and place here?

**Response**: Thank you for question. We looked in to the Plos One format and we didn’t see the significance and removed from the title and affiliation page as suggested.

**Abstract**

Background

1. **Comment**: Line 31-33: “Healthcare workers, being the front line in health delivery system, are expected to play a critical 32 role in cervical cancer screening”. Do the authors believe that health workers should be role models in healthcare services utilization? Instead, it is better to state what makes health workers the major focus in this research? Are they at higher risk?

**Response**: Dear reviewer thanks for the question and the suggestion. In this study, “the healthcare workers, being the front line in health delivery system, are expected to play a critical role in cervical cancer screening”. Yes, the health care workers especially, female healthcare workers are considered to be the role model for cervical cancer screening service utilization in the community. Please refer to the highlighted numbers 96-104 of the introduction section to the details of the description about this specific point with the appropriate supporting references.

2. **Comment:** Line 33: do authors think that saying “Female” is necessary while talking about cervical cancer, no one can consider that males may take this service.

**Response:** Dear reviewer, thank you for the comment. It is true no one can consider males take this service. The word “female” in the above statement was used for the smooth flow and ease of understanding to the readers. However, we don’t have any objection to remove it from the sentence so long as the idea of the statement is upheld.

Method

1. **Comment:** Line 40-42: authors should differentiate the difference between “Bivariate and Bi-variable”, also “Multivariate and Multi-variable”. Please use the correct terms elsewhere in the document.

**Response:** Thanks for the comment and suggestion. Since we are measuring the association of the independent variables, it is appropriate to use the terms, “bivariable and multivariable”. So, we made the revision on line 40-42 and maintained the use of the same terms throughout the manuscript. The revisions are yellow color highlighted.

Result

1. **Comment**: 50-52: better to show the figure

**Response**: Thanks for the comment and the revision has been made as suggested. Line 50-51

2. **Comment**: Line 52-58: please put the AOR immediately after each respective variable.

**Response**: Thank you and the revision has been made as suggested. Line 52-54.

3. **Comment**: Use appropriate spacing and typology.

**Response**: Thank you for the comment and the revision and correction has been made where needed.

4. **Comment**: Line 56: write the VIA in its full word

**Response**: Thank you and the revision has been made as suggested. Line 56

Conclusion

1. **Comment:** What are the criteria to say low?

**Response:** The reference is the World Health Organization cervical cancer elimination strategy. Please refer to the **Line 360-61.** In addition, in developed countries and some developing countries, example like Cameroon (>40%) the screening utilization is higher than that of our study. Line 318

**Introduction**

1. **Comment:** Line 67: better to start with definition, and basic clinical and epidemiologic concepts of cervical cancer.

**Response: As per the comment suggested the necessary revision made. Please refer to line 68-69 and 70-71**

2. **Comment:** Line 99-100: clearly stated the uptake of CCS was low. knowing this, why the authors need additional study? This also contradicts with statement on line 32-33.

**Response:** Dear reviewer as clearly stated by the previous studies done in Ethiopia and Some African countries the cervical cancer screening uptake is reported to be low. The main reason behind considering this study is to explore the screening service utilization among female health workers in Addis Ababa. Addis Ababa as clearly stated in the manuscript, is the capital city of the country where screening service introduced and implemented invariably in all government health institution as of 2020. In addition, the previous studies did not address all female health workers rather have selectively addressed nurses working in one hospital and community health extension workers. Our study on the other hand involves female health workers from the primary health care system (health centers) to tertiary (referral) hospitals and all health- related disciplines with various academic levels. Most importantly, this study is also meant to explore the reasons that hinders or promotes the health workers screening service utilization uptake. We feel the study is rather complementary. Please refer to line 113-117

3. **Comment**: In Ethiopia which age group is a target for CC screening services? Better to introduce the CC screening program in Ethiopia

**Response**: Dear reviewer, Ethiopia has adopted the new WHO cervical cancer prevention and control guideline and implemented an arranged screening to start at age 30 years (targeted age) except for women living with HIV/AIDS where the age to start screening could be lower than the general popupation. Please refer to line 85-93 for the details.

**Method**

1. **Comment:** Line 135-137: write to the point, do not need elaborate extra information unrelated to the subsection. Only study design and period should be stated.

**Response:** Thank you for the comment and necessary revision made as suggested by removing the unnecessary details from the stated lines. Please refer to line 134-135 in the setting section.

2. **Comment**: State eligibility criteria in detail under separate subsection.

**Response**: Dear reviewer thanks for your comment. The eligibility criteria have been clearly stipulated under the study participant section as per the Plos One requirement. Please refer to Line 141-152

3. **Comment:** Line 125: why private health facility workers excluded? Authors should justify**!**

**Response:** Thank you for the question**.** The main reason behind delimiting the study to the public health facilities is to make the study more manageable and feasible within the given time frame and budget. Additionally, as stated in the manuscript, the countries organized cervical cancer screening program which was launched in 2018 and being implemented freely as of 2020 was solely in the public facilities. Moreover, the national organized training program against cervical cancer prevention was given mainly to health workers practicing in these public health institutions. Cervical cancer screening practice in the private health facilities are opportunistic and done by the treating doctors. Please refer to line 95-96 and 135-136

4. **Comment**: Line 133: add details related with cervical cancer screening services and female health workers in the study setting.

**Response**: Dear reviewer, this point has been detailed and addressed in the study participant section. Please refer to line 146-150 of the study participant section.

5. **Comment**: Prior to selection how the authors differentiate those eligible? For example, how did you know their sexual activity? Cervical cancer status? Hysterectomy status? before selection and interviewing?

**Response**: Thank you for the question. Since this is self-administered questionnaire, the participants were briefed before the distribution of the interview about the eligibility criteria and fortunately enough all of the participants approached and recruited during the data collection were eligible for the study.

6. **Comment**: Are healthcare workers and health professionals the same?

**Response**: Thank you for the comment and question. The term “Healthcare workers” is a slightly broader term that includes healthcare professionals and some healthcare supporting staff. Our study participants are all health professionals which could be encompassed in the healthcare workers. Hence, in this study we used healthcare workers and healthy professional interchangeably.

7. **Comment**: Line 152: please state clearly how multi-stage sampling was applied, show each stage, stratifications, sampling units and selection processes

**Response**: Thank you for the suggestion and the comment. Necessary revision made on the manuscript. Please refer to line 155-158. In addition, we have included the flow chart showing the details of the sampling as supporting information, please refer to the uploaded (S1 Fig).

8. **Comment**: Sample size calculation should consider adjustment on marginal error in line with the proportion of the outcome from previous study. Do you think using d=0.05 for p=15% fair? Usually, this margin of error corresponds with high proportion (40-60%)!

**Response**: Thank you for the comment and concern on the margin of error of 5% in the proportion of outcome being 15%. It is clearly stated that as the sample size increases the margin of error decreases. Most authors also stated that at 95% confidence the margin of error falls between 4-8%. In addition, for the proportion (prevalence) outcome between 10-90% the 5%margin of error is the appropriate choice at 95% confidence limit. From the above description the sample size calculation in our case using the margin of error of (0.05) at 95% confidence would yield an adequate sample size for the given proportion(prevalence).

**Comment**: Line 160: why design effect of 2? how many stages do you have?
**Response**: Thank you for the question. There were 4 stages in the sampling process. Please refer to S1 Fig.

9. **Comment**: Line 161: write study variables under separate subsection entitled “Study Variables”

**Response**: The comment incorporated and the revision made as suggested. Please refer to line 169-172

10. Comment: Line 164: show the allocation process clearly.

**Response**: Has been revised as suggested, please refer to line 155-158 and S1 Fig uploaded as the supporting information document.

11. **Comment**: Operational Definitions”, “Data collection Tools and Procedures”, “Data Quality and Management “are missing. Please incorporate them!

**Response:** Dear reviewer, the stated items were not missing. Rather placed under the statistical analysis section to minimize the subheadings. However, if you the reviewer strongly recommend and the Plos One formatting upheld, the material can be addressed under separate subheadings. Accordingly, we have addressed the data collection and quality under one heading and the statistical analysis stands as it stands. Please refer to line 181-194

12. **Comment**: these statements are best suited under data collection subsection. Under data analysis section start your statement from data entry.

**Response**: Dear reviewer, thank you for the comment and suggestion. I agree with your comment and have no objection of revising the subheading as suggested. However, as I stated on the response section of comment 12 above, the only limitation could be the multiple subheadings. I remain beholden to comply with your suggestion and ready to revise as long as it does fit with Plos One formatting requirements. Please refer to line 195-197.

13. **Comment:** Line 184-186: authors should operationalize how they measured knowledge and attitude? Why mean was used?

**Response:** Thank you for the comment and as per your suggestion, operational definition section has been included**. Please refer to line** 393-409.

Mean is commonly used in knowledge assessment questions within research because it provides a single, easily interpretable value that represents the "average" level of knowledge across a group of participants, allowing researchers to compare different groups or conditions and draw conclusions about overall knowledge gain or differences in understanding on a given topic.

**Result**

1. **Comment:** Line 199: how monthly income was categorized? Do you have reference?

**Response:** Thank you for the question and our reference was the World Bank 2020 income-based classification.

2. **Comment:** Line 201: use the standard table and table titles. This comment is applied for all tables**.**

**Response:** Thank you for the comment. We have seriously considered and incorporated your valuable comment. The tables are revised for the clarity, easy to understand by the readers, and the consistency. The titles are also carefully revised to meet the journal requirements.

3. **Comment**: Is there a profession called “HO”

**Response**: Dear reviewer thanks for the question. Yes, “Health officers (HO)” are mid-level health professionals who are trained for four years slightly similar to the nursing profession but with focus on public health track. They are placed in health centers practicing mainly preventive medicine and health center administration. However, they are also placed similar nurses to practice clinical services at health centers.

4. **Comment**: Why “HO” and “IESO” merged elsewhere?

**Response**: Thank for the question. The Integrated emergency surgical officers (IESO) are health officers (HO) who obtained limited surgical training to perform only emergency surgeries, like cesarean section, appendectomy, in the remote parts of the country where the country can’t afford to assign general surgeons and obstetricians and gynecologists.

5. **Comment**: Put specific numbers for “others” under the foot note.

**Response**: The comment acknowledged and revised as advised.

6. **Comment**: Table 2 is not clear. if you need to show response for each knowledge question, write the question and show their responses

**Response**: Thank you for the comment and Table 2 revised as suggested to uphold the clarity.

7. **Comment**: Table 3 and 4 are poor quality and difficult to understand, should be modified to align the quality requirements of the journal.

**Response**: Comment well accepted and major revision made for Tables 3 and 4 to maintain clarity to the information. Please refer to the Tables 3 and 4.

8. **Comment**: Utilization of CC screening should be stated under separate section

**Response**: Comment well accepted and revised as per the suggestion. Please refer to line 277-78.

9. **Comment**: Indicate the interval estimate of the overall CC screening services utilization.

**Response**: Dear reviewer thanks for the comment and question. The overall cervical cancer screening utilization was different for the total study participants and the targeted age group, which was about 19.4% (95%CI 19.36-19.44) and that of the targeted age group (age >=30years) was 31.2% (31.16-31.64). Please refer to the line 259-260.

**Comment**: Line 163: the term Determinant” is not appropriate for this study.

**Response**: Thank you for the comment. We have revised and changed the word “Determinant” by “Factors affecting”. Please refer to line 277

10. **Comment**: Improve the way of reporting findings. Also do not use a long list followed by respectively, line 271-276.

**Response**: Comment accepted and revision made as advised. Please refer to lines 282-87 and294-97

11. **Comment**: Table 6 is poor quality, please improve it. Use consistent font style, color, bolding, size and spacing

**Response**: Thank you for the constructive comment and suggestion. Table 6 has been seriously revised to uphold the quality and the clarity. Please refer to Table 6.

12. **Comment**. The value (sample) in each cell should be adequate to fit the regression model. Unless it will cause numerical error. Please do any measurement to solve these problems. For e.g. marital status single and divorced contain too few values in the cells.

**Response:** Dear reviewer, thank you once again for your constructive comment. A "small sample size" in regression analysis refers to a dataset with too few data points, which can significantly undermine the reliability and validity of the results obtained from the regression model, making it difficult to accurately estimate relationships between variables and potentially leading to misleading conclusions; generally, a good rule of thumb is to have at least 10 observations per independent variable in a regression analysis. The total observations under “divorce” variable in our table is six. However, the smaller sample size (observation) is expected to favor significant association which is not the case in our analysis. Therefore, we have decided not to manipulate the values in the table further but decided to address this under the study limitation section. Please refer to line 377-79.

13. **Comment**: Interpret all statistically significant associations.

**Response**: All statistically significant association were interpreted accordingly in the discussion section.

14. Comment: You can also remove p-values, because CI is better explaining the association.

Response: Thank you for the comment and suggestion. Dear reviewer, I have no objection with your comment. However, there are still some experts and authors in the field who argue and suggest to keep the p-value with the CI. With due respect to your suggestion, we prefer to keep the P-value.

**Discussion**

1. **Comment:** Line 297: use CI of the prevalence to compare your finding with others’.

**Response:** Dear reviewer**,** the prevalence of the screening service utilization was reported from other studies as percentage and ours is not different. So, we feel it is appropriate to compare percentage with percentage of the study participants. However, the CI has been addressed in the result section. Please refer to line 259-60.

2. **Comment:** Line 299: justify the possible explanations for the observed consistency in terms of the findings

**Response:** Please refer to line 313-14 for justification.

3. **Comment:** Line 303-310: how the difference in study design and population could result the observed discrepancy? How your study might differed from a study that included only nurse professionals?

**Response:** Dear reviewer, as clearly stipulated in the methods section, our study is more representative of the female healthcare workers than the studying a subset of the female healthcare workers, “nurses”. Our study also tried to address the observed difference in terms of the primary objectives, which is the screening service utilization among the different female healthcare workers with different levels of education and varying disciplines (from 1^st^ degree to specialties). In addition, our study was done in the capital city of the country involving health professional from the primary level (health centers) to those working in the tertiary facility (referral hospitals. The type and level of exposure to various health related problems is different if someone is working in the hospital or at health centers.

4. **Comment:** Line 311-313: is not clear, “The prevalence of screening utilization among the participants in the targeted age group (age ≥30 years) of 31.2% is slightly high” stands for which study?

**Response:** The comment accepted the statement revised accordingly. Please refer to line 327

5. **Comment**: Line 313-315: the justification is not clear! How difference in primary objective caused the observed difference?

**Response**: Thank you for the comment and the statement has been revised to uphold reasonable explanation. Please refer to line 328-29

6. **Comment**: Line 317-321: do the authors think age difference is the only cause of the observed variation? I think many factors like sociodemographic, economic, health literacy, health setups, etc. Used be considered

**Response**: Thank you for the comment and suggestion. As you have clearly stated difference in the “age” was only one factor considered. Otherwise, the other factors that could contribute to the difference were addressed in the subsequent lines. Please refer to lines 335-337

7. **Comment**: Line 323-357: Interpretations should be stated under result. Under discussion section you need to compare and justify with other studies.

**Response**: Thank you for the comment and suggestion. We have seriously considered the comments given and revised the discussion section as advised. Please refer to Line 350-366

8. **Comment**: Line 327: explain how knowledge helps to increase utilization. Add this explanation for all associated factors with references.

**Response**: Dear reviewer, this point has been detailed in the various sections of the manuscript. As stated in the manuscript; having good over all knowledge about the disease, doctors as the source of the information, knowledge about the risk factors, knowledge of the screening and treatment methods, working in the screening units all have contributed significantly for “knowledge” to have positive impact on the service utilization among the study participants in our study.

**Comment**: Add the policy implication of your finding in Ethiopian context.

**Response**: Dear reviewer this comment has been addressed in the conclusion and recommendation section.

9. **Comment**: The authors should disclose the limitation of the study

**Response**: Thank you for comment and we have addressed the limitation of this study. Please refer to line 371-74

**Conclusion**

1. **Comment:** This section should be based on pertinent findings

**Response:** The conclusion section has been revised as suggested, please refer to line 377-82

2. **Comment**: No need of numerical figures in this section

**Response**: Revision made as per the suggestion. Number removed from the conclusion section. Please refer to lines 377-82 as above

3. **Comment**: Recommendations should consider based on major findings.

**Response**: We have incorporated the comments and revision made as suggested. Please refer to lines 383-386

**References**

1. **Comment:** Check for completeness of all references. For example,
a) ref#3, year of publication repeated**.**

**Response: Thank you for the comment and r**evision made. Please refer to ref number 3

b) Ref#16. is not consistent with Vancouver style.

**Response**: Revision has been made as commented. Please refer to ref number 16

III. Response to reviewer #2

Dear reviewer thank you for constructive comment and guidance.

**Abstract**

1. **Comment: P**lease edit according to journal requirements and world limits. Abstract could be much briefer

**Response:** Dear reviewer thanks for taking your precious time to review this manuscript and providing us with constructive comments. Maximum effort has been made to incorporate the comments to revise the abstract as suggested. Please refer to the abstract section

**Method**

1. **Comment:** Lines 161-164: these lines seem to be irrelevant to the sample size calculation. A dedicated section in the methods is needed to define all study variables and outcomes of interests**.**

**Response:** Thank you for the comment. We have no objection to revise by removing the stated section. However, we are also required to give due details of the sample calculation procedures. We have incorporated your constructive comment and dedicated section to the study variables and outcome variables of interest. Please refer to the methods section

**Result**

1. **Comment:** Lines 274-275: “working in cervical cancer screening units showed an independent association with the screening service utilization”, this statement and finding would not be surprising as working in such centers is a confounder of the cervical cancer screening utilization**.**

**Response:** Dear reviewer thanks for the comments. As clearly stated in the introduction section and the reference materials cited, working in cervical cancer screening unit was reported as one factor affecting cervical cancer screening utilization. We have also considered and studied the place of work as one factor affecting cervical cancer screening utilization. Your comment is reasonable and one may need to consider this comment as an input in future consideration of similar study. To our surprise and as health professionals working on woman cancer, there were many female health professionals who obtained the training on cervical cancer prevention including the screening services and providing the care to others but never dare to get screened. One need to perform a separate subgroup analysis to see if working in cervical cancer screening units stands as one factor affecting screening service utilization or plays a confounding role needs to be investigated.

**Discussion**

1. **Comment:** Please add a paragraph on the study limitations**.**

**Response:** Dear reviewer once again thanks for the comment and we have addressed the limitation. Please refer to the highlighted lines on the paragraph stating strength and limitation
